# *Trypanosoma evansi* and “*Candidatus* Mycoplasma haemolamae” Co-Infection in One-Humped Camel (*Camelus dromedarius*) from the Northwest of Iran: A Case Report

**Published:** 2019

**Authors:** Bijan ESMAEILNEJAD, Aram SAADI, Bahram DALIR-NAGHADEH, Awat SAMIEI, Vahid MOHAMMADI, Ali PIRNEJAD-TALATAPEH, Shahin EHTESHAMFAR

**Affiliations:** 1. Department of Pathobiology, Faculty of Veterinary Medicine, Urmia University, Urmia, Iran; 2. Department of Internal Medicine and Clinical Pathology, Faculty of Veterinary Medicine, Urmia University, Urmia, Iran; 3. Central Laboratory, Faculty of Veterinary Medicine, Urmia University, Urmia, Iran

**Keywords:** Dromedary camel, *Trypanosoma*, *Mycoplasma*, PCR, Iran

## Abstract

A 4-year-old male one-humped camel (*Camelus dromedarius*) was referred to Veterinary Teaching Hospital of Urmia University, Iran in 2017 with anorexia, weakness, depression and pale mucosa. Decreased red blood cell count, packed cell volume and hemoglobin concentration were detected by complete blood cell count. In Giemsa-stained peripheral blood smears *Trypanosoma* spp*.* trypomastigotes scattered between erythrocytic spaces and *Mycoplasma*-like organisms were observed attached to the surface of erythrocytes. Species-specific PCR assay confirmed *T. evansi* and *Candidatus* Mycoplasma haemolamae (CMhl) co-infection. Administration of diminazene aceturate, oxytetracycline 20%, flunixin meglumine and phosphorus-vitamin B_12_ were not effective in treatment. Hemoplasmosis should be considered as an important differential diagnosis of conditions associated with hemolytic anemia in camel.

## Introduction

*Trypanosoma evansi* is a widely-distributed haemoflagellate of veterinary importance that infects a variety of larger mammals including horses, mules, camels, buffalo, cattle and deer and mainly transmitted by hematophagous flies including, *Tabanus* sp., *Stomoxys*sp., *Lyperosia* sp. and *Haematobia* sp. (1, 2). *T. evansi* infection incurs enormous economic losses including mortality, production losses costs of veterinary diagnosis/treatment and growth retardation in camel-raising flocks, throughout the tropical and subtropical regions; including Iran, with prevalence rates between 0% and 19.47% (3–5). It also is implicated as a zoonotic agent, causes of public health problem (2, 6).

Hemotropic mycoplasmas (generally less than 1.0 μm in diameter), formerly known as *Eperythrozoon* or *Haemobartonella*, are small epierythrocytic bacterial agents lacking a cell wall. They are found attached to erythrocytes and may be observed free in the plasma of different species of vertebrates, including rodents, ruminants, pigs, alpaca (*Lama pacos*) and llamas. They are regarded as the most pathogenic species in livestock with clinical signs, especially in those with hemolytic anemia (7). *Candidatus* Mycoplasma haemolama (CMhl) was first described in 1990 in the USA in llamas and reported that CMhl infection alone, contributes to severe hemolytic anemia, fever, jaundice, hemoglobinuria, and weight loss in camelids (8). The present report describes the first case of a hemolytic disorder in an Iranian one-humped camel (*Camelus dromedarius*) co-infected with *T. evansi* and CMhl from northwest of Iran.

## Case Presentation

In Jul 2017, a 4-year-old male one-humped camel (*Camelus dromedarius*) from Salmas county, Urmia, Iran belonging to a flock composed of 70 animals was referred to the Veterinary Teaching Hospital of Urmia University, Iran with poor body condition and signs of anorexia, weakness, depression and pale mucosa. According to the farmer, the symptoms started 5 d ago. The camels were reared in the pasture and only brought into the paddock during the night. The flock had no history of vaccination, anti-parasitic treatment, insecticides application or any other medication was recorded. Rectal temperature of the camel was 39.6 °C, heart rate was 50 beats per min and respiratory rate was 15 breaths per min. The body of camel was inspected carefully for the presence of ectoparasites. Jugular blood samples were taken into vacutainers (Kendall Company, Covidien, USA) containing EDTA-K2 as anticoagulant for determination of hematological and molecular analyses. During examination, the whole body of camel was examined for the presence of ticks by palpation, mainly on their ears, along their nape of neck, perineum, and udder/orchid, between thigh, shoulder region and tail base. The ticks were manually removed and transferred to the parasitology laboratory in tubes containing 70% ethanol solution.

**Fig. 1:**
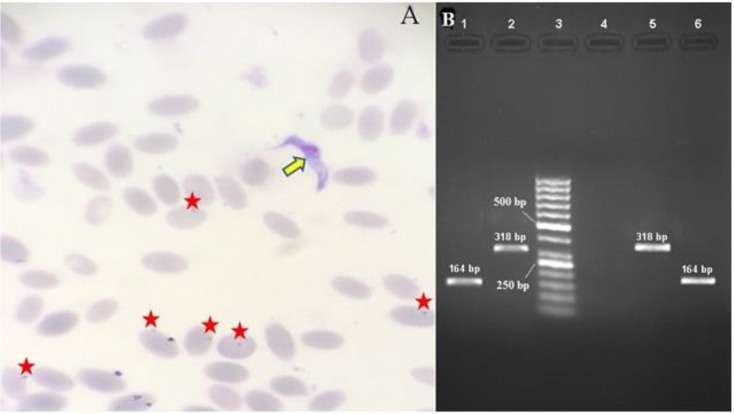
A) *Trypanosoma evansi* (arrow) and epierythrocytic “*Candidatus* Mycoplasma haemolamae” attached on the surface of camel red blood cells (stars) (Giemsa, 100×). B) Amplified products using *T. evansi* and epierythrocytic “*Candidatus* Mycoplasma haemolamae” specific primers. Lane 1: *T. evansi* infection; Lane 2: “*Candidatus* Mycoplasma haemolamae” infection; Lane 3: 50 bp DNA ladder; Lane 4: Negative control; Lane 5: *Candidatus* Mycoplasma haemolamae positive control; Lane 6: *T. evansi* positive control

Light microscopic examination of Giemsa stained peripheral blood films revealed the presence *Trypanosoma* spp. trypomastigotes (20×2.2 μm) and *Mycoplasma*-like organisms (up to 0.5 μm) (Fig. 1A). *Mycoplasma* spp. appeared as small, coccoid, basophilic epicellular bodies freely available in plasma or epicellular attached to the surface and periphery of erythrocytes.

Reduced number of RBCs (3.25×10^6^/μl, reference interval: 6.38±0.38×10^6^/μl), elevated total leukocyte count (37×10^3^/μl, reference interval: 12.38±0.97×10^3^/μl) and decreased hemoglobin concentration (7.4 g/dl, reference interval: 12.00±0.63 g/dl) and low packed cell volume (PCV) (21%, reference interval: 37.21±2.48%) were found in hematological examination (9). Genomic DNA was extracted from 25 μL whole camel blood using a commercially available kit (Thermo Fisher Scientific, Dreieich, Germany) according to the manufacturer’s instructions and stored at −80 °C until further use. A pair of primers, CMhl -F 5′- TAG ATT TGA AAT AGT CTA AAT TAA -3′ and CMhl -R 5′- AAT TAG TAC AAT CAC GAC AGA ATC -3′ were used to amplify a 318 bp fragment of the ssu rRNA gene of CMhl*.* The primer’s specificity and sensitivity were assessed (10). PCR was carried out in 50 μl total reaction volume containing 5 μl of 10 x PCR buffer, 2 mM MgCl_2_, 250 μM of each of the four deoxynucleotide triphosphate, 1.25 U Taq DNA polymerase (Fermentas, Germany), 50 pmol of each primer and 50 ng of extracted DNA. Amplification of parasite DNA was done in thermocycler CP2-003 (Corbett Research, CP2-003, Australia). Cycling condition for *Candidatus* Mycoplasma haemolamae was 94 °C for 10 min, followed by 32 cycles at 94 °C for 60 sec, 50 °C for 60 sec and 72 °C for 2 min with a final extension step of 72 °C for 70 min. “*Candidatus* Mycoplasma haemolamae” positive control confirmed by GenBank under accession number MF356308. According to the method (11), using a commercially prepared specific primer set (TBr1& TBr 2) that yields a 164 bp product for the specific detection of *T. evansi*. Primer sequences were as follows: TBr1- 5′-GAA TAT TAA ACA ATG CGC AG-3′, TBr2- 5′-CCA TTT ATT AGC TTT GTT GC-3′. PCR amplification reaction was carried out in 1x buffer containing 50 mM KCl, 10 mM Tris-HCl pH 8.3, and 0.1% Triton X-100, enriched with 1.5 mM MgCl 0.2 mM of each dATP, dCTP, dGTP and dTTP (Promega, USA), 25 pmol each primer, 50 ng of genomic DNA and 1.0 U of *Taq* DNA polymerase (Promega, USA). All components were mixed and sterile distilled water was added to a final volume of 50μl. PCR reactions were conducted in thermocycler CP2-003 (Corbett Research, CP2-003, Australia) programmed for an initial denaturation and activation step at 94 ºC for 10 min. This step followed by 40 cycles of 94 ºC for 30 sec (denaturation), annealing step at 50 ºC for 45 sec and extension step at 72 ºC for 60 sec. Post-extension was by one cycle at 72 ºC for 10 min followed by holding at 4 ºC. *T. evansi* positive control (kindly provided by parasitology division of the Bu-Ali Sina University under accession number KR184820 (Fig. 1B).

Treatment included subcutaneous injection of diminazene aceturate (Aburaihan Co., Tehran, Iran; 7.5 mg kg^−1^), intramuscular injections of oxytetracycline 20% (Aburaihan Co., Tehran, Iran; 0.2 ml kg^−1^) and flunixin meglumine (20 ml, Aburaihan Co., Tehran, Iran). Furthermore, phosphorus-vitamin B_12_ (Aburaihan Co., Tehran, Iran; 0.5 ml kg^−1^) were prescribed as supportive care subcutaneously. The treatment was relatively efficient in remission of clinical signs for only three days and the animal died 10 d after initiation of treatment.

Twelve adult ticks were collected that the most frequent and abundant tick species found on camel were *Rhipicephalus sanguineus* and *Hyalomma annatolicum annatolicum.*

## Discussion

Previous studies from various southern regions of Iran employing PCR reported positive cases of *T. evansi* camels varying from 1.6% to 25.75% (2, 12–15). However; this study is the first molecular identification of causative agent of surra in north-west of Iran. The disease is usually chronic and characterized by fever, anorexia, listlessness, pale mucous membranes, dullness, a very thin hump and drop to one side, abortions in pregnant females, and death in untreated camels (16–18).

Although, infection of camelids by hemomycoplasma has been described previously in camels (*Camelus dromedarius*) from central and south regions of Iran (Yazd and Kerman Provinces) using cytologic and molecular examination (19, 20); however, to the best of the authors’ knowledge the present report is the first documented and confirmed case of CMhl infection in Iranian one-humped camels from northern half of Iran. Anemia is a pathognomonic finding of surra and CMhl infections in the camelids. Decreased number of RBCs, hemoglobin concentration and PCV confirmed the occurrences of anemia in this case. These results are in agreement with earlier findings (19, 21). This report was remarkable in several aspects I) molecular identification of *T. evansi* and *CMhl* co-infection in camel from Iran, II) association of hemoplasma infection with pyrexia and intravascular hemolysis in an in one-humped camel. Since ticks and hematophagous insects are important sources of *Trypanosomes* and CMhl infections in this area, it appears that such infections may be common in camels. The common clinical signs of both infections are fever, pale mucosa, and extremities edema. Since the hemomycoplasmiosis is a secondary infection, any kind of stressful, immune suppressing and retarding condition could aggravate the disease.

Rational treatment would appear to consist of correcting the anemia and eliminating of the infection. However, recovery was not achieved by administration of diminazene aceturate, oxytetracycline, and phosphorus-vitamin B12.

## Conclusion

Hemoplasmosis should be considered in the differential diagnosis of diseases characterized by hemolytic anemia and pyrexia. The PCR evaluation for hemoplasma DNA should be included in the investigation of such cases to enable the rapid definitive detection of this infection, which may be more common than previously estimated. Conducting further case studies are necessary to recommend successful treatment.
